# Are the Effects of Variation in Quantity of Daily Bilingual Exposure and Socioeconomic Status on Language and Cognitive Abilities Independent in Preschool Children?

**DOI:** 10.3390/ijerph17124570

**Published:** 2020-06-25

**Authors:** Raffaele Dicataldo, Maja Roch

**Affiliations:** Department of Development and Socialization Psychology, University of Padova, 35131 Padova, Italy; maja.roch@unipd.it

**Keywords:** socioeconomic status, bilingual exposure, language development, cognitive development, preschool children

## Abstract

Bilingual exposure (BE) and socioeconomic status (SES) are associated with children’s development, but their specific and unique effects are still unclear. This study analyzed the influence of these environmental factors on a set of cognitive and linguistic abilities in preschoolers to disentangle their effects. One hundred-eleven Italian-speaking preschool children (mean age = 61 months; SD = 6.8) growing in a monolingual or multilingual context completed an assessment of cognitive (theory of mind, inhibition, attention shifting and working memory) and linguistic abilities (vocabulary, grammar, narrative comprehension, lexical access). The results of hierarchical regressions with predictors variation in BE (both Length and Daily exposure) and SES on each ability, shown a specific contribution of variation in SES, after controlling for BE, in vocabulary, grammar, and working memory (WM), and a specific contribution of variation in BE, over and above effect of SES, in vocabulary, narrative comprehension and WM. In addition, we found an interaction between these factors in predicting the performance of the theory of mind task (ToM). To conclude, variations in BE and SES are related independently to individual differences in linguistic and cognitive skills of children in preschool.

## 1. Introduction

According to a bioecological theoretical framework [1], child development is a transactional process in which an individual’s development is influenced by his or her interactions with various aspects and spheres of their environment. Most measures of the ‘environment’, such as parenting, social support, and life events, show significant genetic influence [2], but genetics are just one piece of the intricate puzzle that makes up a child’s life. 

Individual differences in children’s language and cognitive skills are strictly related to early life experiences, in addition to a variable degree of genetic influence, that can have a profound impact on the developing brain, its organization [3,4] and its adaptation to the environment [5]. 

Bilingual exposure (BE) and socioeconomic status (SES) are two environmental factors that have been found to be linked to children’s developmental trajectories and to the variation in their linguistic and cognitive profiles [6]. 

Several studies tried to identify the ways in which these two environmental factors are associated with children’s development, however, identifying the unique and specific role of BE experience and SES on children’s linguistic and cognitive outcomes is particularly difficult because of the frequent co-occurrence of these factors that produces confounding effects [7]. The purpose of the present study is to examine the specific effects of variations in BE and SES to determine how much each factor is related to linguistic and cognitive outcomes in preschoolers and to analyze whether their effects are interactive or additive.

### 1.1. Effects of Bilingual Exposure on Language and Cognitive Abilities

The term Bilingual exposure refers to the linguistic experience in which a speaker is regularly exposed to more than one language in everyday life [8]. The definition of bilingual exposure is more complex than a “yes/no” categorization, however, to date there is no standard definition of bilingual exposure or a common standard for determining how to describe individuals in terms of this complex experience [9]. 

The main source of difficulty in conducting research with children experiencing such complex language experience, is assessing language exposure and obtaining reliable estimates of daily, weekly, and overall distribution of the input languages in heterogeneous groups of children [10]. 

Generally, the measures of bilingual exposure that have been used are length of exposure, cumulative exposure and daily exposure. Length of exposure is a coarse measure that allows one to measure the lifespan exposure of a specific language and is operationalized as the children’s chronological age, i.e., age at time of testing, minus their age of onset, i.e., age at time of first exposure to a language. The measure of cumulative exposure suggested by Unsworth [11], instead, was intended to capture the sum of bilingual children’s exposure to language over time. This measure takes into account: (i) how much each parent and any other adults living in the home spoke each language for each one-year period in the child’s life to date; and (ii) whether the child attended daycare or school in these periods, and if so, what the language of instruction was there. This information makes it possible to calculate the proportion of each one-year period, which includes exposure to each language spoken, and summed up, gives the total amount of exposure to languages in years over time. Finally, daily exposure is a measure to estimate the percentage of exposure to each language during each day, providing detailed information about the time spent talking in one language and in the other(s). 

Exposure to more than one language early in life has the potential to yield structural changes in the brain [12], changes in the patterns of neural activation during language processing and, eventually, changes in children’s language and cognitive skill development [13,14]. The extant literature provides robust evidence that age of onset, length of exposure, cumulative exposure and daily exposure are important source of variability for linguistic and cognitive development [11,15,16,17,18].

Thus, since the categorical distinction does not take into account individual differences in language experience, the field should move away from monolithic bilingual vs. monolingual comparisons [19]. As stated by Luk and Bialystok [20], language experience lies on a continuum: individuals are not categorically bilingual or monolingual. The authors found that bilingual experience was best represented through multiple factors in the statistical model. In a heterogeneous bilingual sample, bilingual experience was shown to involve at least two dimensions: bilingual usage on a daily basis, and language proficiency in one of the two languages. These recent findings echo those of Fishman and Cooper who found that self-reported language proficiency and usage were the best predictors for four linguistic ratings of bilingualism [21], suggesting that bilingual experience is best represented by multi-dimensional measurements.

According to this recent literature, in this work, rather than defining children as monolinguals or bilinguals, we describe their exposure as monolingual or bilingual using two continuous variables that take into account children language experiences: length of exposure and daily exposure (both defined on a continuum).

Differences in the amount of exposure to the majority language (i.e., Italian) are mainly due to the fact that the children participating in the study come from both monolingual and multilingual contexts. We decided to combine children who develop in monolingual and bilingual contexts since we were interested to examine whether even a small difference in length of exposure to the majority language and in daily exposure to the majority language are related to preschoolers’ performance in cognitive and linguistic skills. 

Studies conducted to date indicated that the length of exposure is a crucial factor in determining differences in language development. The strongest effect of BE on language development concerns vocabulary growth. Children coming from multilingual families receive less exposure to each language than children coming from monolingual families because their parents need to divide language input between two languages [22]. 

The quantity of language exposure has a significant influence on the size of children’s vocabularies across the age range from 30 to 60 months, with the result that the children with multilingual exposure lag behind monolingual children on measures of both receptive and expressive vocabulary in each spoken language [23,24,25,26]. Additionally, evidence suggests that the length of exposure to the majority language is also linked to morpho-syntactic development and language proficiency [22,27,28,29]. 

Currently, an increasing number of studies have addressed the role of bilingual exposure on narrative skills in preschoolers. These studies have shown that macrostructural knowledge in narrative production and comprehension is invariant across both languages [30]. Moreover, even children with a limited expertise in one of the two languages, are able to produce stories with an adequate narrative structure and a good global quality [31,32]. 

Research conducted to date showed that individual differences in all the linguistic aspects mentioned above are strictly dependent on the amount of BE. 

A parallel body of research has examined the effects that BE has on cognitive abilities. Nowadays there is large debate about the so-called “bilingual advantage” which refers to research findings demonstrating that bilingual exposure has positive effects on cognitive control components that fall under the umbrella term “executive functions” (EFs) [7,33,34,35,36]. 

Bilingual exposure may promote EF advantages because children manage multiple languages and continuously monitor the appropriate language for each communicative interaction [37]. However, the existing literature on whether there are differences in the efficiency of EF functioning linked to the bilingual exposure, particularly in children, reported mixed results. On one hand, some research with preschool and early school-aged children has shown that bilingual exposure promotes better performance in attentional shifting [34], response inhibition [38], inhibitory control [7,39], and cognitive flexibility [40,41]. 

On the other hand, numerus studies consistently failed to find any significant difference in performance in cognitive tasks linked to bilingual exposure [42,43]. In addition to the results reported on EF, also findings in the research area focused to determine whether bilingual exposure promote better performance in theory of mind-related tasks (ToM) are inconsistent. Goetz found that bilingual exposure promotes better performance in the ToM-related tasks [44], whereas in a more recent study conducted by Dahlgren and colleagues [45], earlier results in several ToM tasks were not replicated [45]. 

Recently, more controlled studies have argued that advantages linked to bilingual exposure found in many published papers may be due to hidden demographic factors, namely SES and ethnicity, and not to bilingual exposure per se [38,43,46]. Variability in familial SES has, in fact, been identified as a potentially important contributory factor in studies reporting cognitive advantages due to bilingual exposure [46,47]. 

Another important aspect in this field is the “publication bias” against studies showing null or negative effects. De Bruin, Treccani, and Della Sala in 2015 [48], found that studies that show an advantage on executive function tasks linked to bilingual exposure are more likely to be published than studies that show mixed results, null results, or a disadvantage, and reported that only 29% of the studies that showed no effects of bilingual exposure or even a disadvantage were published. The presence of hidden factors, often not controlled, in previous studies and the existence of this publication bias have likely led to a misrepresentation of the effect that bilingual exposure has on cognitive development. Finally, previous studies mostly have compared pre-selected groups of bilinguals and monolinguals, and this may have influenced the results. Moving towards a definition of bilingual exposure on a continuum that not only considers bilingual and monolingual exposure but also quantifies it in terms of time (lifespan) and daily experience could bring to light new answers on the effects of linguistic experience on linguistic and cognitive development. 

### 1.2. Effect of SES Variation on Language and Cognitive Abilities 

SES, typically measured as a combination of parental education level, employment status, occupation prestige, and household income [49], is correlated with a broad range of important life outcomes, such as intelligence, academic achievement and school readiness of kindergarteners [50,51]. Although there is likely to be some degree of genotype–SES correlation, i.e., a child’s genotype is associated with parental SES, this association is not purely genetic, as environmental influences are likely to contribute too [2], and in this paper we focused our attention mostly on these influences.

Early studies in this field generally used a between-group design comparing children of middle and working class [52]. However, recent studies using SES as a continuous variable demonstrated that even small differences in SES may explain individual differences in linguistic and cognitive skills, suggesting that it is preferable to use a continuous variable in order to better examine the effect that SES has on linguistic and cognitive development [53]. The effect of the variation in SES has been attributed to a generally impoverished linguistic input. As the SES varies, we can find differences concerning the nature of the interaction between caregivers and children, the quantity of speech to which the child is exposed and the nature of that speech [54].

Previous studies suggest that variations in socioeconomic status are correlated with oral language abilities on measures of language processing, comprehension, and production, from infancy through high school [55]. The strongest relationship between the variation in SES and language development is with vocabulary size [56,57]: as the SES decreases, children show a lower vocabulary size, and this effect produces a gap concerning vocabulary development [52]. This gap in vocabulary is already evident as early as the age of 18 months, and by age the of 24 months it presents a six-month disadvantage [58]. Morpho-syntactic skills and narrative competence were also found to be influenced by the variation in SES [55,59]. Although there are no data on the effect of the variation in SES on narrative comprehension, few studies that addressed narrative production show that as the SES decreases, children produce narratives less sophisticated in terms of topic coherence and independence from the nonlinguistic context [60]. 

The effect of the variation in SES is not limited to the verbal domain, as it also influences the cognitive domain [61,62]. Numerous studies report SES-related disparities on composite or latent measures of executive functions (EF) in children as young as 2–5 years old [63,64,65,66]. While there is consistent evidence that suggest that as the SES decreases children perform worse in tasks related to inhibitory control, executive attention, flexibility, and planning [67,68,69], previous findings are inconsistent regarding WM. There are studies that show that the variation in SES affects verbal short-term memory capacity [70], while other findings show no effect of the variation in SES [71]. 

Another construct of human cognition that caught attention for its potential relationship with SES is the theory of mind (ToM). To date, mixed findings are reported concerning this relationship [72,73]. 

It is worth highlighting that the majority of the previous studies in this filed have compared pre-selected groups of children coming from low and high-SES families [52,55,58], therefore further work is needed to obtain a clearer representation of the effects of the variation in SES on linguistic and cognitive development in preschoolers.

### 1.3. The Current Study: Independent and Combined Effects of the Variations in BE and SES

Variations in BE and SES have been shown to influence language and cognitive development in children, but rarely have they been studied jointly. To our knowledge, only four studies have attempted to evaluate independent and combined effects of BE and SES on linguistic and cognitive skills in children [6,7,74,75]. These studies provide the first findings that indicate that each of these experiences contribute uniquely to development, irrespective of the other factor [B]. The lack of interaction between SES and bilingual exposure suggests that SES similarly affects bilingual and monolingual children, and that bilingual exposure similarly affects children from low- and mid–high-SES families [6].

Previous studies often provide scant information concerning the amount of bilingual exposure; most of them have used different measures to determine SES and adopted different tasks to assess language and cognitive skills. Finally, these studies adopted different methodologies to answer this research question. The majority of these studies used a between-subjects design comparing the performance of children exposed to more than one language to demographically matched monolingual peers. Although such designs can establish fundamental group differences in their performance, they are largely insensitive to variability within each group of participants and variability within each classroom. Even a small variation in these variables, as we saw, has the potential to give rise to differences in linguistic and cognitive skills. Moreover, from a strictly statistical perspective, the dichotomization of quantitative measures has substantial negative consequences in most circumstances in which it is used such as loss of information about individual differences, loss of effect size and power or spurious statistical significance [76]. For all these reasons, we moved away from a classic comparison between monolingual and bilingual and between high SES and low SES, considering both environmental variables as a continuum. 

The current study is expected to deepen our understanding on how variations in BE and in SES affects linguistic and cognitive abilities in preschool children. 

### 1.4. Research Questions and Predictions

The main aim of this work concerns the analysis of the specific and unique role of the variation in BE on the one hand, and in SES on the other, in language (vocabulary, grammar and narrative comprehension, lexical access), ToM and cognitive skills (inhibition, shifting and WM) in order to disentangle their role in preschoolers’ development. In addition, the interaction between the two factors is verified. Based on previous studies, it is expected that the two factors play independent, rather than interactive influence in language and cognitive development [6,7,74,75]. We predicted that the variation in SES influences language skills and cognitive abilities, irrespectively of variations in BE, with better outcomes associated with an increase in SES, which would be in accordance with previous literature [7,75].

On the other hand, we expect that language abilities are related to variations in the length of BE, irrespectively of variations in the family SES, as shown in previous studies, with lower linguistic competence associated with the variation in the length of BE [6]. Finally, as studies on variations in BE’s role in cognitive abilities and ToM have shown mixed results, we did not put forward any prediction in this regard.

## 2. Materials and Methods

### 2.1. Participants

This study was approved by the local ethics committee of the University of Padova (protocol number: 2064) and conducted according to the Declaration of Helsinki. One hundred and fifteen children aged between 44 and 75 months recruited from four schools situated in the metropolitan area of Padua—a medium-size city in Northeastern of Italy—participated in this study. We contacted the city’s School Services Department and requested to be informed of the schools in the area in order for participating children to be of very different backgrounds, SES, multilingual exposure and language experience. Based on the information provided, we selected 4 schools in 4 different neighborhoods that were not already involved in other research projects. We presented the research project to school principals who voluntarily decided to allow research in their schools, promoting the project to parents. All children of each class and their families were invited to take part to the study: the group was deliberately heterogeneous in terms of SES and language or languages exposure in order to capture the variability of children who were involved in the school system in the area at the time in which the research was carried out. Parents were asked to sign a consent form if they agreed to take part and let their child to take part. Parental permission forms were distributed to parents of all children of the age range 4–6 years in the selected schools. We handed out 150 consent forms and received consent for 115 children, 111 of whom (61 boys; 50 girls) were included into the study (mean = 61.9 months, SD = 6.8 months). One child was excluded for hearing problems declared by parents and 3 for their incomplete assessment.

### 2.2. Materials and Procedure

Parents filled in a questionnaire at home and returned it to school. Children were tested individually in three sessions, in a quiet room in school. All tasks were presented to children in Italian, i.e., the language of context, which for children from bilingual family corresponded to second language (L2). Tasks were presented in a fixed-order and each session lasted approximately 30 min.

#### 2.2.1. Background Measures

Parents filled in a detailed questionnaire consisting of 40 items divided into 4 sections [77,78]. For this study, we used data of two sections to compute the two independent variables, namely, SES and BE (see Appendix A):

Demographic and socioeconomic status: We measured SES as the education level of both parents and the annual family income level. In both cases, data were collected categorically: parental educational levels were classified into 6 categories (1 = primary education degree, 2 = middle school degree, 3 = high school degree, 4 = bachelor’s degree, 5 = master’s degree, and 6 = post-graduate education) and annual income was coded based on a 5-point scale (1 = below EUR 24,000, 2 = EUR 25,000–30,000, 3 = EUR 31,000–34,000, 4 = EUR 35,000–40,000, 5 = above EUR 40,000). Categories were transformed into a continuous scale of years of education and Euro (in thousands). A composite SES score was calculated by combining income and education levels into one variable through principal components analysis [79]. For each participant, mother’s education, father’s education, and household income are entered into the model, and a composite SES score is computed.

Language status and use—we used the following variables concerning the language status and bilingual exposure:

(a) Onset age of language exposure to L2 (Oa): Starting from onset of exposure to L2, we computed the length of exposure to the language of context (hereafter, LELC) as the difference between age of onset and age at the time of testing. This continuous measure provides the amount of time that they spent, in their whole life, exposed to the language of context, that while for monolingual children corresponds to chronological age, for children exposed to more than one language may vary in length.
(b) Daily amount of input of each language: we asked parents to indicate where and with whom the child spend time on an average day in a week and for how long, and which language(s) each person used when addressing the child, using a ten-point scale (passive input). At the same time, we asked parents to estimate which language(s) the child used to answer to that person using a ten-point scale (active input). We computed the variable daily exposure to the language of context (hereafter, DELC) as a continuous measure, given by the mean percentage of the current daily use of each language in different contexts and with different persons. For monolingual children, this percentage corresponds to 100, and for children exposed to more than one language, it ranges between 0 and less than 100%.

#### 2.2.2. Linguistic Skills

Receptive vocabulary (Peabody Picture Vocabulary Test-Revised; PPVT—R): The PPVT-R is a standardized test for receptive vocabulary [80]. It consists of a list of words in order of increasing difficulty and is presented to participants who are asked to indicate which of 4 pictures best represents the target word. A basal-level is defined based on the child’s ability to give 8 consecutive correct answers. Testing is then continued until the participant obtains 6 incorrect answers out of the last 8 words presented (ceiling-level). Raw scores correspond to the number of correct answers minus the number of errors (range 0–175). Standard scores are computed based on raw scores: M = 100, SD = 15. Reliability, evaluated using the split-half procedure, as reported in the test manual, is 0.88.

Semantic access (speeded naming NEPSY-II subtests): Speeded naming subtest from the linguistic domain of NEPSY-II [81], was used to obtain normed measures of rapid semantic access and production of colors, shapes, and sizes. The tester shows to the child an array of colors and shapes; colors, shapes, and sizes; or letters and numbers and asks them to name them in order as quickly as possible. For each item, accuracy, self-corrections and speed are recorded. Scaled scores that combine time and accuracy were calculated (mean = 10, SD = 3). Test–retest reliability is 0.93 in the youngest children (3–4 years) and 0.72 for children between 5- and 6-years old.

Grammar comprehension (PVCL): The Prova di Valutazione della Comprensione Linguistica (Test for the Evaluation of Linguistic Comprehension) is a standardized test for children aged 3–6 and 8 years which evaluates grammar comprehension [82]. Sentences contained salient morpho-syntactic cues, such as gender and number agreement, conjunction, negation, different types of phrasal structures (i.e., relative, passive, temporal). Children were required to choose which picture from among a set of four correctly represented the sentence spoken by the experimenter. One point was credited for each correct answer and the percentage of correct answers was the total raw score. Raw scores can be converted into weighted scores ranging from 0 to 100; these scores evaluate children’s overall performance taking into account the number of correct answers and also the level of difficulty of each item. 

Narrative comprehension (TOR 3–8): The test TOR 3–8 is a standardized test for Italian language, which evaluates narrative comprehension of children aged between 3 and 8 years of age [83]. The tester read two stories aloud and, to minimize the cognitive and memory load, he/she interrupted reading at two predetermined points and asked multiple-choice comprehension questions. The tester presented four alternative answers, both verbally and using pictures, and asked participants to point to the correct picture. Comprehension of each story is assessed using 10 questions, half concerning information explicitly stated in the story and the other half requiring inferences to be generated. The score consists of the sum of correct answers, 10 for each story, with a maximum score of 20. Raw scores are transformed into standard scores with the mean = 10, SD = 2. The internal reliability, evaluated by calculating Cronbach’s alpha over items, ranges from 0.52 to 0.72.

#### 2.2.3. Cognitive Skills

Working memory (digit span task): digit span, Wechsler Intelligence Scale for Children (WISC) sub-test [84], was used to assess WM. Tester reads aloud a list of pre-determined random numbers ranging from two to nine digits in the forwards trial and from two to eight digits in the backwards trial. In the forwards trial, children were asked to repeat the digit sequence in the same order. In the backward trial, that requires simultaneous storage and processing of information in memory, children were asked to repeat the sequence in reverse order. Th digit span score corresponds to total number of trials, forwards and backwards, completed correctly (range 0–32). The longest list lengths correctly repeated in the two trials were reported as forward and backward spans (forwards = 9; backwards = 8). Reliability, evaluated using the split-half procedure, is 0.81.

Executive functions (FE-PS 2–6): Two subtest of FE-PS 2–6, namely the day and night test and the dimensional change card sort were used [85].

The Day and Night test was used to assess inhibition, the ability to suppress a dominant response related to perceptual stimuli in the task while selecting and executing a competing, conflicting response [86]. This task contains two decks of cards: the first contains 8 cards depicting a chessboard and 8 an X; the second deck contains 8 cards depicting a sun and 8 a moon. In the control condition, the tester trains the child to say “day” when there is a X card and “night” when there is a chessboard card. In the inhibition condition (i.e., the Stroop condition) the child has to say the word ‘day’ when viewing a card depicting a nighttime sky and ‘night’ when shown a picture of the daytime sky. Each child completed 16 trials for each condition that were scored 0 (incorrect) or 1 (correct). Three different scores are calculated: accuracy (range 0–16), speed (in seconds), and inhibition, which is given by subtracting the accuracy in the inhibition condition from performance in the control one (range −16 to 16). The reliability, evaluated by calculating Cronbach’s alpha over the items, is 0.85.

The dimensional change card sort (DCCS) was used to assess attention-shifting skill. This task involves sorting neutral cards based on characteristics of the object presented on the cards. The DCCS consists of 3 decks of cards (one for each phase) and requires that children sort each card presented into 1 of 2 locations/piles according to a rule provided by the experimenter. During the first phase (“shape game”), children are instructed to sort cards into the correct piles based on shape. In the second phase, children are told that the rule has changed and they now must sort cards based on color (“color game”). In the third phase (“border game”), children are told that cards with a border would be sorted according the role of the “shape game”, while cards without a border would be sorted according the role of the “color game”. The tester records how many cards the child classify correctly in each trial: performance in the shape and color version are scored as the number of correct choices out of 6; performance in the border version is scored as the number of correct choices out of 12 (range accuracy 0–24). Total accuracy was used. Test–retest reliability is 0.36.

Theory of mind (ToM): We developed a contents false belief task adapted from a previous study [87]. In this task, children were shown a “pasta box” and were asked to guess its contents. Then, the tester showed the actual contents of the box (i.e., pencils) and asked children to identify what the object was. In the control trial (“self” question) the tester asked: “What did you think was there when you saw it?”. The second part of this task involves the ToM trial: another person comes into the room for a while, looks at the “pasta box” and then leaves; after this, the tester asks the child what the other person thought would be in the box (“other” question). The score ranged between 0–2: either 1 (correct) or 0 (incorrect) is given to the “self” and “other” question.

## 3. Results

### 3.1. Descriptive Statistics

#### 3.1.1. Socioeconomic Status

The group varied widely in socioeconomic status (see Table 1): years of education ranged from 5 to 22 for the mothers and from 5 to 20 for the fathers, with a mean of 13 years being equivalent to receiving a high school degree (SD = 3.3); household income ranged from EUR 18,000 to over EUR 41,000, with a mean of EUR 30,340 (SD = EUR 8.317), which is equivalent to the Italian median family income [88].

A positive moderate correlation between the education level of both parents (r = 0.44, *p* < 0.001) was found. Moreover, income and education levels were positively related to one another (0.40 < r < 0.51 *p* = 0.001) and thus were combined into one continuous variable (SES) using principal components analysis [79]. For each participant, mother’s education, father’s education, and household income were entered, and a composite SES score was computed. The first principal component weighted education and income equally and accounted for 64 percent of the original variance. The mean score of the composite is 0 (SD = 1). Families with high scores on the SES composite variable have high annual income levels and a high level of education.

#### 3.1.2. Language Status and Use

We found that for 28 families, there was at least one parent whose native language was not Italian. In detail, we found that *N* = 10 were Romanian speaking, *N* = 8 Chinese-speaking, *N* = 3 Arabic-speaking, *N* = 3 Moldavian-speaking, *N* = 2 Russian-speaking, *N* = 1 Albanese, *N* = 1 Turkish-speaking. The majority of children coming from these families were born in Italy and only two children were born abroad. All the children raised in these families have been receiving significant continuous exposure to both languages as of before the age of 3 years, and have received at least 2 years of formal language learning provided in educational settings. 

The definition of bilingual exposure that we adopted in this study reflects the typical situation of many classrooms in Italy where children are classified as Italian learners as an additional language if a different language is spoken at home or in their community [89]. This classification designated 83 children as exposed to only the language of context and 28 varying in bilingual exposure. This number corresponds to 25% of the group, a percentage higher than that found in the last national survey, according to which, in the area of our study, the percentage of children exposed regularly to more than one language during preschool was 15% [89].

The group varied widely also in exposure to the language of context (L2 = Italian). Concerning the age of first exposure to Italian, we found that the range of onset age (Oa) was very wide: from 0 month to 36 months (mean = 6.3; SD = 10.1 months). Starting from this information, we computed the length of exposure to the language of context (LELC) which ranged between 24 and 75 months. The ranges of current daily exposure to the language of context (DELC) and the daily language use were very wide. Current daily exposure to the language of context (DELC) ranged between 29 and 100, whereas children’s daily language use ranged between 33 and 100.

In addition, we ran two logistic regression analyses to predict the probability that the two continuous measures used to assess the bilingual exposure better discriminate between children growing in a monolingual context (exposed from birth to the language of context, 100% of the daily time) and peers growing in a bilingual context (being exposed at least for 24 months to more than one language and for at least 50% of time to each language during the day). In the first one, the predictor was the length of exposure to the language of context (LELC). A test of the full model versus a model with intercept only was statistically significant, 2 (32, *N* = 111) = 52.207, *p* = 0.014. The model was able to classify correctly 42% of those who were classified as children exposed to more than one language and 98% of those who were classified as exposed only to the language of context, with an overall success rate of 84%. In the second logistic regression, the predictors were length of exposure to the language of context (LELC) and daily exposure to the language of context (DELC). A test of the full model versus a model with intercept only was statistically significant, 2 (50, *N* = 111) = 125.3, *p* < 0.001. The model was able to classify correctly 100% of children exposed to more than one language and peers exposed to one language. These two analyses suggested that to assess correctly the bilingual exposure, LELC is not sufficient and DELC is needed in addition. Therefore, in the following analyses, we decided to use these two continuous variables as separate indicators of the variation in bilingual exposure (BE).

#### 3.1.3. Language and Cognitive Skills

All participants, except one child, completed all the tasks (descriptive statistics in Table 2). Performance in the majority of tasks covered a large range of scores and none suffer from the ceiling effect. Distributions of the majority of the variables approached symmetric, with the exception of performance in the DCCS task. An inspection of frequencies of scores indicated that the large kurtosis value was because 75% of children scored between 16 and 21. 

Concerning receptive vocabulary, our group lay at the lower boundary of the range appropriate for age. Standard deviation was comparable to that of the national standardization sample, therefore, we concluded that the range of performance was typical and unrestricted. The children’s performance in semantic access and narrative comprehension were appropriate for age. Concerning grammar comprehension, the performances of the children show a good variability. The grammar comprehension task (PVCL) allows one to place the performance of children in one of 7 “classes of merit” calculated by the scores’ distributions of standard samples of different age. The majority of the children in our group—40%—show a below-average level of performance, 36% an average performance and 24% an above-average performance. Performance in cognitive tasks covered a large range of scores showing a good variability. None of cognitive tasks suffered from either floor or ceiling effects (only one child obtained the highest score on DCCS). In the ToM task, 14% of the children obtained 0, 49% obtained 1 and 27% obtained 2.

### 3.2. Correlations between SES, BE and Performance in Linguistic and Cognitive Tasks

As can be seen from Table 3, the variation in length of exposure (LELC) and daily exposure to the language of context (DELC) shows a weak to moderate correlation (0.19 < r < 0.55) with all linguistic and cognitive outcomes. The greatest correlation (r = 0.55, *p* < 0.001) was, as expected, between the variation in DELC and receptive vocabulary. In regard to SES, we found a moderate correlation with receptive vocabulary (r = 0.44, *p* < 0.001), grammar and narrative comprehension, WM and ToM. A pattern of significant correlations emerged among language and cognitive skills. 

### 3.3. Specific Correlation between the Variation in Bilingual Exposure and SES with Linguistic and Cognitive Skills

To identify how much the variations in daily BE and in SES are associated with linguistic and cognitive skills in preschoolers, a series of hierarchical regression analyses were carried out on scores obtained in each task. Predictors added in the first step were the variation in length of exposure to the language of context (LELC), daily exposure to the language of context (DELC) and SES were added in the second and third step, and the interaction between DELC and SES in the fourth step. For each measure, two hierarchical regression analyses were carried out in which the order of entry of DELC and SES (second and third steps) was inverted, while the remaining predictors were left invariant. This procedure allowed us to assess unique variance accounted for by each predictor controlling for the other. In other words, we looked at the correlation of the variables added in the third step. 

To summarize, these are the models’ specifications: 

In Model 1, we aimed to analyze the specific effect of the variations in SES. In the first step, the variation in length of exposure was added, then in the second step, the variation in current exposure was added. In the third step, the variation in SES was added, and in the last, the interaction. In Model 2, we aimed to analyze the specific effect of the variation in current exposure on the language of context. In the first step we entered the variation in length of exposure, then the variation in SES, followed by the variation in current exposure and finally the interaction. 

Table 4, Table 5, Table 6, Table 7, Table 8, Table 9, Table 10 and Table 11 show the specific contribution of differences in SES (Model 1) and the variation in DELC (Model 2) with variations in length of exposure to the language of context (LELC) and interaction, which are elements that both models share. Table 4, Table 5, Table 6, Table 7, Table 8, Table 9, Table 10 and Table 11 show the results of the analyses with adjusted R^2^, β coefficients, and significance levels.

The results revealed that the models, including the variation in BE and in SES, and their interaction explain a 6–78% range of total variance in various linguistic and cognitive skills. The highest amount of variance is explained for vocabulary, narrative comprehension and WM (R^2^: 78, 0.22 and 0.33, respectively). For all measures, except for semantic access and ToM, the variation in length of exposure to the language of context (LELC) correlated with the highest amount of total variance of both linguistic and cognitive skills: the longer the exposure to the language of context, the higher the linguistic and cognitive outcomes. In particular, for linguistic skills, it explains 28% of variance in receptive vocabulary, 8% and 17% in grammar and narrative comprehension, respectively, whereas for cognitive skills, it explains 18% of variance in WM, 7% in inhibition and 10% in attention shifting. 

**Figure 1:**
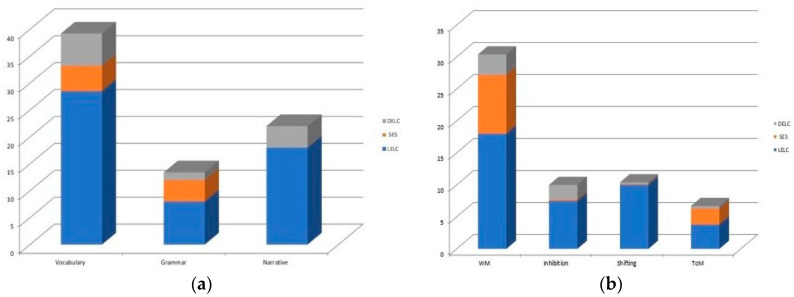
The percentage of unique variance explained by variation in SES (Model 1) are reported: (**a**) specific effect on linguistic skills; (**b**) specific effect on cognitive skills.

**Figure 2:**
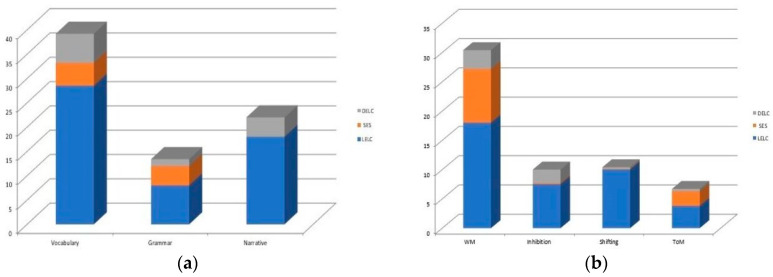
The percentage of unique variance explained by variation in BE (Model 2) are reported: (**a**) specific effect on linguistic skills; (**b**) specific effect on cognitive skills.

Independent and specific effects of the variations in both SES and in DELC when added to the third step and after the entrance of the other variable were found in linguistic and cognitive skills. The variation in SES accounted from 4% to 9% of unique variance in several cognitive and linguistic abilities, indicating a stronger influence than that of the variation in DELC. The children’s performance in receptive vocabulary, grammar comprehension and working memory is higher with a greater SES condition. On the other hand, when the variation in DELC is added to the third step, it correlates specifically with the children’s outcomes in receptive vocabulary (6% of unique variance) and narrative comprehension (4% of unique variance) more so than the variation in SES, with higher performance associated with greater daily exposure to the language of context. The variation in DELC correlated significantly with the performance in WM (3% of unique variance), which is higher in the function of greater daily bilingual exposure than the influence of the variation in SES. There is only one significant interaction that emerged between the variation in SES and the variation in DELC, which was in ToM performance. This shows that better outcomes were found for children of higher SES more exposed to the language of context. These results are also reported in Figure 1 and Figure 2. 

## 4. Discussion

The aim of this study was to investigate the specific contribution of two environmental factors, namely, BE and SES, on a large set of cognitive and linguistic skills (specifically, vocabulary, grammar, narrative comprehension, lexical access, theory of mind, inhibition, attention shifting and working memory) and to analyze whether their effects were interactive or independent.

Although we believe that genetic contributions should also be considered when dealing with associations between parents’ behavior and their children’s behavior [90], in this study we focused our attention on the environmental influence that SES and BE have on child development. As reported in previous sections, individual differences in parental SES, which is defined primarily in terms of educational attainment, occupation and income, are only partly genetic [91]. 

In this study, participants come from the same metropolitan area in which Italian is the language of context, but differed in levels of parental education, primary language spoken, length of exposure and daily exposure to Italian. Few studies have explored the independent and combined effects of variations in BE and in SES on a large set of preschoolers’ cognitive and linguistic skills. Furthermore, in most of the previous work, children were tested in English as L2. One strength of this study is that all the participants are viewed as forming a continuum of language exposure and SES patterns rather than as representing discrete groups, i.e., monolingual versus bilingual, high versus low SES, etc. As argued by De Bruin [92], regarding bilingual exposure, despite bilinguals and monolinguals being typically treated and compared as two uniform and distinct groups, it is very rare that even two children are the same since they may have different experiences and immersion in a bilingual environment. All children, regardless of their first language (L1), were tested with same tools on linguistic and cognitive abilities in the language of context. This allowed us to quantify the effects of the variation in SES and in the experience with more than one language.

The main finding of this work is that both a variation in bilingual exposure and in SES are associated with individual differences in a large number of linguistic and cognitive skills during preschool age, and their correlations are independent and specific. In particular, we found a specific correlation between the variation in SES, after controlling for the variation in BE, with vocabulary, grammar and working memory. On the other hand, the variation in BE predicted specifically vocabulary and narrative comprehension more effectively than the variation in SES. Furthermore, WM was specifically associated with the variation in BE, but the association was of an opposite trend when compared to the association between BE and linguistic skills. In fact, a higher working memory was associated with greater BE. Finally, an interaction between the two factors in predicting theory of mind was marginally significant, suggesting that a higher ToM is associated with a condition of higher SES and greater exposure to the language of context. We failed to find specific effects of the variation in both SES and BE on rapid naming, inhibition and attention shifting. 

Research investigating the effect of environmental factors on language and cognitive outcomes has led to the awareness that the life situation of each child, in addition to genetic and heritable factors, is uniquely complex and that life experiences might not be wholly independent of each other [8]. The current work provides new evidence that the variation in the experience related to the two environmental factors examined contributed specifically to language and cognitive skills, irrespective of the other factor. The specific contribution of each factor is discussed in the following sections.

### 4.1. The Role of the Variation in SES in Language and Cognitive Skills of Chidren of Preschool Age

Previous studies consistently demonstrated a negative effect of low SES on language development, indicating that the impact of SES was equivalent for both children exposed to one or more languages [7]. The current findings are in line with previous literature. However, we took the investigation into the role of the variation of SES in language and cognitive development a step further. In the current study, we did not compare discrete groups defined as belonging to low and high SES; rather, we considered the SES variable on a continuum, which has the advantage of capturing individual differences among participants without categorizing them within a specific group. SES variations accounted specifically for 4–9% of variance in receptive vocabulary, grammar comprehension and in working memory. Interestingly, we failed to find a specific influence of the variation in SES on narrative comprehension and some cognitive skills (inhibition, attention shifting and ToM). Rather than taking this result as evidence that these tasks are free from SES influence, we argue that this influence might not be evident for this age range or that the tasks used are not sufficiently sensitive to capture this influence, or even that this relationship is not specific. Future studies will have to clarify this point. The amount of variance in language and WM explained specifically by the variation of SES might appear small. Nonetheless, these significant contributions suggest that even subtle variations in SES, irrespective of the linguistic and cultural background, may affect children’s developmental trajectories. 

In line with previous studies, it was demonstrated that a lower SES is not only associated with the verbal domain: living in underprivileged backgrounds, which provide fewer and less adequate social-cognitive stimulation, is related also to children’s cognitive abilities [58,61,62]. In particular, results of the present study indicated that SES variations are paralleled by variations in working memory (measured through a forward and a backward digit span). WM measures a cognitive capacity that is usually associated with language development, and perhaps this association reflects a more general correlation between WM and language [93]. This possibility should be further examined in future studies, given that WM is essential for a variety of linguistic, cognitive and learning skills. A negative impact of SES condition may put children with lower SES at risk regarding a variety of abilities related to school readiness and later learning success.

### 4.2. The Role of the Variation in Bilingual Exposure in Language and Cognitive Skills of Children of Preschool Age

One of the most original contributions of this work concerns the way in which we defined the variable bilingual exposure. Rather than treating it as a dichotomous variable that distinguishes between bilinguals and monolinguals, we defined BE as: (1) a continuous variable (ranging from 100% of monolingual exposure to 50% of balanced exposure in each language) and (2) a multidimensional variable consisting of two different measures: length of exposure to the language of context which defines the linguistic exposure at life experience level and the estimate of daily input (both active and passive) to each language that the child is exposed to, which defines their current experience with languages. These two variables together allow us to classify correctly 100% of the group according to their bilingual exposure. In addition, these measures allow us to capture the role of more subtle variations in BE without categorizing participants as bilinguals, or not taking into account all the linguistic experiences. Moreover, these variables capture all the variations in BE in the functions of the quantity of life and daily exposure to all the languages spoken. 

The main source of difficulty in conducting research with children experiencing such complex language experience, is assessing language exposure and obtaining reliable estimates of daily, weekly, and overall distribution of the language input in heterogeneous groups of children [8]. There are several measures used to assess bilingual exposure. For example, language exposure has been measured by recording direct language spoken to a child at home during the course of a day and calculating the amount of exposure to each language [94]. A more common and efficient approach is to measure language exposure on the basis of parent reports in terms of a daily diary for several days or through a questionnaire assessing exposure across the lifespan [95,96]. Other assessments include the amount of language exposure reported from each conversational partner as rated on a scale [97,98], and others simply ask parents to estimate the percentage of exposure to each language [11,99]. Thus, language exposure assessments vary in terms of the tools used to assess it (e.g., direct language input or parent report), and the time period assessed (e.g., one day, several days, or the entire lifespan). Many of these measures are quantitative and some also consider the quality of language interactions. All these differences have an effect on the results reported in the literature, and make a straightforward and comparable interpretation difficult.

We used a similar methodology already adopted in some previous studies trying to define the complexity of bilingual exposure [11]. We took a step further in examining the specific role of the variation in BE conceived in this way in a variety of linguistic and cognitive skills. Our group was therefore deliberately heterogeneous with the purpose of capturing the variability of children who are currently considered as bilingual in preschool classrooms in Italy, and capitalizing on the notion of bilingual exposure as a continuous measure.

The length of exposure to the language of context, which indicates the precocity of potential bilingual exposure, explained the highest amount of variance in all the tasks considered ranging from 7 to 28% of variance. This means that bilingual exposure should occur as earlier as possible in a child’s life, and later onset of bilingual exposure is associated with a negative impact on a large set of linguistic and cognitive skills, especially during first phases of bilingual exposure. Our data support this claim by showing that the longer the bilingual experience, namely, the length of exposure to the language of context, the higher the outcomes in language and cognitive skills (with the exception of WM). 

The specific influence of the variation in current exposure, namely, the daily amount of linguistic input, explained 3–6% of variations in linguistic comprehension (vocabulary and narrative comprehension) and in WM, independently from the variation in SES. Lower outcomes in vocabulary and narrative comprehension are obtained in association with greater bilingual exposure. In line with previous works, children more exposed to the language of context outperformed children who are less exposed to the language of context in language comprehension and production [26]. This finding was unsurprising, given that usually children who are exposed to more than one language simultaneously are not able to devote as much time to each of their language as they would if they were learning only one. Children exposed to more than one language tend to have smaller vocabularies in both languages compared to their peers and less sophisticated narrative comprehension [100,101]. However, this study brings novel and more specific insights into this relationship regarding children exposed to more than one language because of the detailed documentation of these children’s amount of exposure to the language of context (quantity of exposure) and because we were able to control other confounding factors. In addition, to our knowledge, this is the first time that influence of the variation of BE in narrative comprehension was addressed and highlighted. Future studies will have to confirm this finding providing more systematic and empirical evidence, taking into account also measures of quality of interaction between parents and children in order to obtain a more detailed measure of language exposure, namely, quantity and quality of exposure.

On the other hand, children’s performance in WM was higher in functions of greater BE. Perhaps continuous daily experience of exposure to more than one language requires a greater memory load, and this enhances the working memory. We would like to highlight that our participants were 4–6 years old children: this WM benefit could well transfer in later stages of development to other cognitive skills; also, this benefit might represent a resource for language learning in children with bilingual exposure. Finally, we would like to outline that the 3% of variance, explained by the variation in BE, is independent from any influence exerted by the variation in SES: in other words, the same benefit of bilingual exposure characterizes children with different SES levels. Few studies have investigated the possibility that bilingual exposure also affects the working memory (hereafter, WM). Bialystok and colleagues in 2004 and Morales, Calvo and Bialystok in 2013 [74,75], found some fragmentary evidence of a bilingual advantage on WM. However, other studies comparing simple working memory performances have found no evidence for significant differences due to monolingual and bilingual exposure [93,102,103]. 

Our findings are not completely in line with the study of Meir and Armon-Lotem [6], in which they failed to find evidence for the independent effect of BE on a forward span task. However, in our study, we have a measure of the backward span task as well as the forward one, which indicates a more active aspect of WM. It might be speculated, expecting further confirmation, that bilingual exposure affects specifically the active components of WM. We argue that the procedure and stimuli used to test working memory were chosen appropriately to be equally familiar to all children because of very well-known items (digits). Consequently, this task might be unlikely relevant to yield significant advantages or disadvantages in children differing for language knowledge and experience. As reported above, there is a need of further studies in which different tasks for the assessment of different aspects of WM should be adopted.

Beside WM, we failed to find any significant specific contribution of the amount of daily bilingual exposure on executive functions and ToM, suggesting that we do not have any evidence supporting cognitive advantages due to bilingual exposure. In our study, measures of attention shifting and inhibition, together with ToM, appear to be neither enhanced nor attenuated by bilingual exposure. This result is in line with a large corpus of studies whose results are at odds with prior evidence of cognitive advantages due to the fact that bilingual exposure has challenged the generality of the effect. Paap, Johnson, and Sawi (2015) argue that cognitive advantages are a mere artifact of experiments, namely, either do not exist, or are restricted to specific aspects of bilingual experience that enhances only specific components of EF, such as non-linguistic cognitive control [104,105]. Our results are in line with those of Morton and Harper [46], suggesting that by controlling for the variation in SES, the advantage previously found for children exposed to more than one language becomes attenuated. Certainly, this is not a definite conclusion, given that this is a small study; rather, it represents a new starting point on which to build new knowledge on the existence of and the actual amount of cognitive advantages linked to the bilingual exposure.

## 5. Limitations and Future Purposes

Although the current investigation provides evidence about the specific effects of variations in SES and in BE, the study is not without limitations.

First, the sample size is too small to be highly informative. Our study was indeed a trial project specifically for testing whether considering the variables SES and language exposure as continuous variables produces noteworthy findings. We acknowledge the need to collect further data that can give more strength to these preliminary results. Future studies with a larger sample size are needed to disentangle the specific effect of SES and BE on linguistic and cognitive skills in preschool children in order to better understand their combined effects. Our findings, in line with previous studies, suggest that the variation in SES similarly affects children who experience bilingual exposure and children who are exposed to only one language, and that the variation in BE affects similarly children of different SES levels. An exception is provided by the marginally significant interaction between variations in SES and BE that emerged from the performance in the ToM task, showing that higher ToM is paralleled by increasing SES and more exposure to the language of context; conversely lower ToM is associated with a lower SES and a higher amount of BE. The small sample size of our work, however, does not allow us to draw firm conclusions. There is a need for further studies with much larger samples to obtain more robust results in order to refute the existence of interactions between SES and BE.

Secondly, some of the study assessment tools used to measure bilingual exposure and cognitive skills differ from the tools used in previous literature, and this limit us in comparing our results to the literature. Concerning the assessment of bilingual exposure, we decided to use a relatively new questionnaire—currently under validation—because it contains the variables that we needed to measure properly the exposure to language in terms of quantity and SES. Future research, while maintaining a multidimensional bilingual exposure assessment, should consider the use of variables that evaluate also qualitative aspects of language input and interaction. For instance, it would be interesting to evaluate other relevant sources of language exposure, namely television and video, frequency of literacy activities at home and school, and so on, that may provide additional information.

Concerning the tolls used to assess cognitive skills and, in particular, WM, we used the digit span task to assess it. Although it is not uncommon to use the digit span test in order to measure working memory capacity, the problem with it is that it does not thoroughly examine both the storage and processing elements of the working memory. Despite this limitation, which we recognize, we prioritized in our study a measure that we were sure that also young children would be able to complete. Indeed, our results are promising in suggesting some benefits of BE in WM; therefore, it is absolutely necessary to plan and conduct further studies in which different tasks for the assessment of different aspects of WM should be adopted.

Finally, in this study we did not assess children’ proficiency in L1. Since this variable may mediate the effect of BE on linguistic and cognitive skills, future studies could also consider this variable as an element of individual variability.

## 6. Conclusions

The present study is the first to assess independent and combined effects of the variations in both BE and SES on a large set of linguistic and cognitive skills in 4–6-year old children, considering the two independent variables as continuous rather than discrete factors, in order to capture the complexity and the variability that characterize both the environmental factors. Although we know that the sample composition of this study was not large enough to draw a definite conclusion about the specific effect of bilingual exposure and SES on language and cognitive development, we believe that it may represent a new starting point to study, in greater detail and accuracy, the effect that bilingual exposure, together with SES, has on children development. 

Even with a small number of children, new evidence for the distributed impact of variations in BE and SES on the linguistic and cognitive development of preschool children has been shown; furthermore, these environmental factors affect different abilities of children, yielding variation in their linguistic and cognitive profiles. More important, it provides new evidence, even if small and nonconclusive, in support for independent effects of variations in BE and SES, on linguistic and cognitive skills in preschoolers and supports existence of some benefits to WM of children exposed to more than one language. 

Variations in BE and in SES influence, at least in part, different skills. While grammar is affected only by the variation in SES and narrative comprehension is predicted solely by variations in BE, vocabulary and WM are affected by variations in both factors, independently. When both factors affect certain skills in the same direction, as in the case of vocabulary, it means that children who have a lower SES and experience a bilingual exposure are more vulnerable, since their performance is affected by more than one environmental factor. On the other hand, when both factors affect certain skill but with an opposite trend, as in the case of working memory, it is desirable that the WM benefits from the variations in BE have a stronger impact than the negative impact of the variation in SES: at least, this should be one of the promising purposes of interventions targeting low-SES children.

Although we acknowledge that the current work has to be improved in strength, the are some relevant theoretical and practical implications. Theoretically, there are conclusions that support the proposal that variations in BE and SES independently affect trajectories of cognitive and linguistic development, pointing to the role of the proximal learning environment. The present work, although with its limitations, suggests that any type of learning environment matters, and the present results demonstrate that even subtle variations in different factors may act through different mechanisms, and largely independently of each other, to shape children’s linguistic and cognitive development. A deeper understanding of the effects of both SES and BE so they can be used to improve the developmental outcomes of all children is needed.

Practically, these findings have implications for the assessment of children exposed to more than one language and coming from different socioeconomic status families, emphasizing the need to consider contextual factors both in the assessment and during educational intervention planning. Direct and indirect interventions may improve learning environments of all children, but we need to learn more about what kind of intervention should be implemented with preschoolers in order to make all children, regardless their family environment, ready to learn at school and eventually to prevent later difficulties.

Finally, we strongly believe that these results provide relevant insights concerning the use of a multidimensional measure to assess language exposure. A multidimensional measure of language exposure, which considers both quantitative and qualitative aspects, allows us to obtain more information about the language experience of each child. It is well known that time and opportunity to hear and use a language influence children’s development and their performance in language and cognitive skills [6,7], thus, the field should move away from monolithic bilingual vs. monolingual comparisons [28] that do not take into account individual differences in language experience and, instead, use variables that are more precise and more informative regarding each child’s language exposure.

## Figures and Tables

**Table 1:** Descriptive statistics of SES measures.

Variable	*N*	Percentage	Range	Mean (SD)
Years of Maternal Education			5–22	13.4 (3.4)
Less than High school degree	22	22.6		
High school degree	39	40.2		
More than High school degree	26	37.2		
Years of Paternal Education			5–20	12.9 (3.5)
Less than High school degree	25	26		
High school degree	44	45.8		
More than High school degree	27	28.2		
Household Income			18,000–46,000	30,340 (8.3)
below EUR 30,000	36	37.1		
EUR 31,000–34,000	34	35.1		
Above EUR 35,000	27	27.8		

**Table 2:** Descriptive statistics (range, mean, standard deviation, skewness and kurtosis) for age and performance in linguistic and cognitive tasks.

Variable	Min	Max	Mean	SD	Skewness	Kurtosis
Linguistic skills						
PPVT-R: Receptive vocabulary (M = 100; SD = 15)	53	118	82.6	13.4	0.32	–0.34
Speeded naming: Semantic access (M = 10; SD = 3)	4	16	9.69	2.8	–0.18	–0.56
PVCL: Grammar comprehension (range: 0–100)	11	93	55	19.2	0.13	–0.73
TOR 3-8: Narrative comprehension (M = 10; SD = 2)	7	15	10.5	1.8	–0.10	–0.73
Cognitive skills						
Digit span: Working memory (range 0–32)	2	15	7.5	2.9	0.03	–0.82
Day and Night: Inhibition (range −16–16)	–11	14	1.8	4.4	0.91	1.9
DCCS: Attention shifting (range 0–24)	7	24	18.4	2.9	–1.01	3.8
ToM: Theory of mind (range 0–2)	0	2	1.2	0.68	–0.30	–0.81

**Table 3:** Correlation matrix of bilingual exposure, SES and linguistic and cognitive measures.

	1.	2.	3.	4.	5.	6.	7.	8.	9.	10.	11.
1. Length (LELC)	1	0.32 **	0.15	0.53 **	0.20 **	0.25 **	0.42 **	0.43 **	−0.26 **	0.30 **	0.19 *
2. Daily (DELC)		1	0.46 **	0.55 **	0.27 **	0.33 **	0.42 **	0.11	−0.21 **	0.04	0.24 **
3. SES			1	0.44 **	0.19	0.31 **	0.24 *	0.31 **	−0.06	0.02	0.22 *
4. Receptive vocabulary				1	0.48 **	0.50 **	0.68 **	0.45 **	−0.27 **	0.20 *	0.45 **
5. Semantic access					1	0.26 **	0.41 **	0.38 **	−0.09	0.26 **	0.30 **
6. Grammar comprehension						1	0.46 **	0.43 **	−0.07	0.27 **	0.39 **
7. Narrative comprehension							1	0.34 **	−0.26 **	0.15	0.33 **
8. Working memory								1	−0.20 *	0.39 **	0.38 **
9. Inhibition									1	0.01	−0.01
10. Attention shifting										1	0.11
11. Theory of mind											1

Note: LELC: length of exposure to the language of context; DELC: daily exposure to the language of context; ** Correlation is significant at the 0.01 level (two-tailed); * Correlation is significant at the 0.05 level (two-tailed).

**Table 4:** Results of the hierarchical regression analyses on receptive vocabulary (PPVT—R). R² = 0.78.

Models	Predictors	R^2^ Change	β	t	*p*
	LELC	0.284 *	0.523	6.099	0.000
Model 1	SES	0.047 **	0.245	2.876	0.005
Model 2	DELC	0.060 ^†^	0.290	3.253	0.002
	SESxDELC	0.001 ^††^	−0.135	−0.269	0.788

* F change (1, 94) = 37.198, *p* < 0.001; ** F change (1, 92) = 8.269, *p* < 0.05; ^†^ F change (1, 92) = 10.580, *p* < 0.05; ^††^ F change, not significant (n.s.).

**Table 5:** Results of the hierarchical regression analyses on semantic access (speeded nam.). R² = 0.06.

Models	Predictors	R^2^ Change	β	t	*p*
	LELC	0.038 *	0.195	1.917	0.058
Model 1	SES	0.015 *	0.136	1.206	0.231
Model 2	DELC	0.000 *	0.093	0.793	0.430
	SESxDELC	0.001 *	0.198	0.287	0.775

* F change, n.s.

**Table 6:** Results of the hierarchical regression analyses on grammar comprehension (PVCL). R² = 0.13.

Models	Predictors	R^2^ Change	β	t	*p*
	LELC	0.079 *	0.281	2.822	0.006
Model 1	SES	0.038 **	0.218	2.037	0.045
Model 2	DELC	0.014 ^†^	0.139	1.242	0.217
	SESxDELC	0.003 ^†^	0.375	0.573	0.568

* F change, (1, 93) = 7.956, *p* < 0.05; ** F change, (1, 91) = 4.150, *p* < 0.05; ^†^ F change, n.s.

**Table 7:** Results of the hierarchical regression analyses on narrative comprehension (TOR). R² = 0.22.

Models	Predictors	R^2^ Change	β	t	*p*
	LELC	0.176 *	0.419	4.479	0.001
Model 1	SES	0.006 **	0.086	0.839	0.404
Model 2	DELC	0.041 ^†^	0.238	2.237	0.028
	SESxDELC	0.000 **	0.008	0.013	0.990

* F change, (1, 94) = 20.058, *p* < 0.001; ** F change, n.s.; ^†^ F change, (1, 92) = 5.006, *p* < 0.05.

**Table 8:** Results of the hierarchical regression analyses on working memory (digit span). R² = 0.30.

Models	Predictors	R^2^ Change	β	t	*p*
	LELC	0.180 *	0.424	4.539	0.000
Model 1	SES	0.092 **	0.343	3.405	0.001
Model 2	DELC	0.032 ^†^	−0.210	−2.002	0.048
	SESxDELC	0.000 ^††^	−0.141	−0.239	0.812

* F change, (1, 94) = 20.605, *p* < 0.001; ** F change, (1, 93) = 4.008, *p* < 0.05; ^†^ F change, (1,92) = 11.591, *p* < 0.001; ^††^ F change, n.s.

**Table 9:** Results of the hierarchical regression analyses on inhibition (day and night). R² = 0.10.

Models	Predictors	R^2^ Change	β	t	*p*
	LELC	0.075 *	0.273	2.753	0.007
Model 1	SES	0.002 **	−0.055	−0.488	0.627
Model 2	DELC	0.023 **	0.179	1.531	0.129
	SESxDELC	0.000 **	0.619	0.944	0.348

* F change, (1, 94) = 7.579, *p* < 0.05; ** F change, n.s.

**Table 10:** Results of the hierarchical regression analyses on shifting (DCCS). R² = 0.11.

Models	Predictors	R^2^ Change	β	t	*p*
	LELC	0.107 *	0.326	3.331	0.001
Model 1	SES	0.001 **	−0.003	−0.028	0.978
Model 2	DELC	0.003 **	−0.063	−0.549	0.978
	SESxDELC	0.006 **	−0.541	−0.802	0.452

* F change, (1, 93) = 11.097, *p* < 0.001; ** F change, n.s.

**Table 11:** Results of the hierarchical regression analyses on theory of mind (ToM). R² = 0.10.

Models	Predictors	R^2^ Change	β	t	*p*
	LELC	0.038 *	0.194	1.907	0.060
Model 1	SES	0.025 *	0.176	1.562	0.122
Model 2	DELC	0.004 *	0.070	0.610	0.534
	SESxDELC	0.040 **	1.35	2.014	0.047

* F change, n.s.; ** F change, (1, 90) = 4.058, *p* < 0.05.

## Appendix A

Items of parents’ questionnaire:

Socioeconomic status:

How many years did you go to school? (indicate the number of years starting from primary school)?

How many years did you go to school? (indicate the number of years starting from primary school)?

What is your educational qualification? (Options: Primary education degree, 2 = Middle school degree, High school degree, Bachelor’s degree, Master’s degree, and Post-graduate education)

What is your husband/wife or partner’s educational qualification? (Same options)

Please indicate the annual income of your household (Options: below EUR 24,000, between EUR 25,000–30,000, between EUR 31,000–34,000, between EUR 35,000–40,000, above EUR 40,000).

Language status and use:

At home, in addition to the language of origin, do you speak Italian? (Options: Yes; No)

Which language(s) do you use with your child? (Options: Italian; language of origin; both)

At what age did the child start hearing Italian (indicate in years and months)?

At what age did the child begin to hear the language of origin (indicate in years and months)?

Who are the people who spend a lot of time at home with your child? In what language do they communicate with the child? In what language does the child respond? First of all mark the role of the person (e.g., mother, father) in both tables and then mark with a vertical line the amount of time (an estimate) that the person addresses the child in Italian or in the language of origin and how much the child answers in Italian or in the language of origin. The vertical line delimits the amount of communication that takes place in Italian (to the left of the line) and in the language of origin (to the right of the line).

**Table A1:** Language experience with different people (indicate your role).

How much does the person speak to the child in which language?											
Italian											Language of origin
How much does the child answer in which language?											
Italian											Language of origin
